# IL-23 past, present, and future: a roadmap to advancing IL-23 science and therapy

**DOI:** 10.3389/fimmu.2024.1331217

**Published:** 2024-04-15

**Authors:** James G. Krueger, Kilian Eyerich, Vijay K. Kuchroo, Christopher T. Ritchlin, Maria T. Abreu, M. Merle Elloso, Anne Fourie, Steven Fakharzadeh, Jonathan P. Sherlock, Ya-Wen Yang, Daniel J. Cua, Iain B. McInnes

**Affiliations:** ^1^ Laboratory for Investigative Dermatology, The Rockefeller University, New York, NY, United States; ^2^ Department of Medicine, Division of Dermatology and Venereology, Karolinska Institute, Stockholm, Sweden; ^3^ Department of Dermatology and Venereology, Medical Center, University of Freiburg, Freiburg, Germany; ^4^ Evergrande Center for Immunologic Diseases, Brigham and Women’s Hospital, Harvard Medical School, Boston, MA, United States; ^5^ Allergy, Immunology & Rheumatology Division, Center for Musculoskeletal Research, University of Rochester Medical School, Rochester, NY, United States; ^6^ Division of Gastroenterology, Department of Medicine, University of Miami Leonard Miller School of Medicine, Miami, FL, United States; ^7^ Janssen Scientific Affairs, LLC, Horsham, PA, United States; ^8^ Janssen Research & Development, LLC, San Diego, CA, United States; ^9^ Immunology Global Medical Affairs, Janssen Pharmaceutical Companies of Johnson & Johnson, Horsham, PA, United States; ^10^ Janssen Research & Development, LLC, Spring House, PA, United States; ^11^ Kennedy Institute of Rheumatology, University of Oxford, Oxford, United Kingdom; ^12^ College of Medical, Veterinary and Life Sciences, University of Glasgow, Glasgow, United Kingdom

**Keywords:** IL-23, cytokine, immune-mediated inflammatory diseases, psoriasis, psoriatic arthritis, inflammatory bowel disease

## Abstract

Interleukin (IL)-23, an IL-12 cytokine family member, is a hierarchically dominant regulatory cytokine in a cluster of immune-mediated inflammatory diseases (IMIDs), including psoriasis, psoriatic arthritis, and inflammatory bowel disease. We review IL-23 biology, IL-23 signaling in IMIDs, and the effect of IL-23 inhibition in treating these diseases. We propose studies to advance IL-23 biology and unravel differences in response to anti–IL-23 therapy. Experimental evidence generated from these investigations could establish a novel molecular ontology centered around IL-23–driven diseases, improve upon current approaches to treating IMIDs with IL-23 inhibition, and ultimately facilitate optimal identification of patients and, thereby, outcomes.

## 1 Introduction

Interleukin (IL)-23 is a member of the IL-12 cytokine family and is implicated in a cluster of immune-mediated inflammatory diseases (IMIDs) that includes psoriasis (PsO), psoriatic arthritis (PsA), and inflammatory bowel disease (IBD; namely Crohn’s disease [CD] and ulcerative colitis [UC]) (1–8). Because of its ability to reprogram immune cells in inducing and maintaining a highly proinflammatory state, IL-23 can be considered a dominant regulatory cytokine that acts as a rheostat, or a master switch, for initiating development and maintenance of these IMIDs.

**Figure 1:**
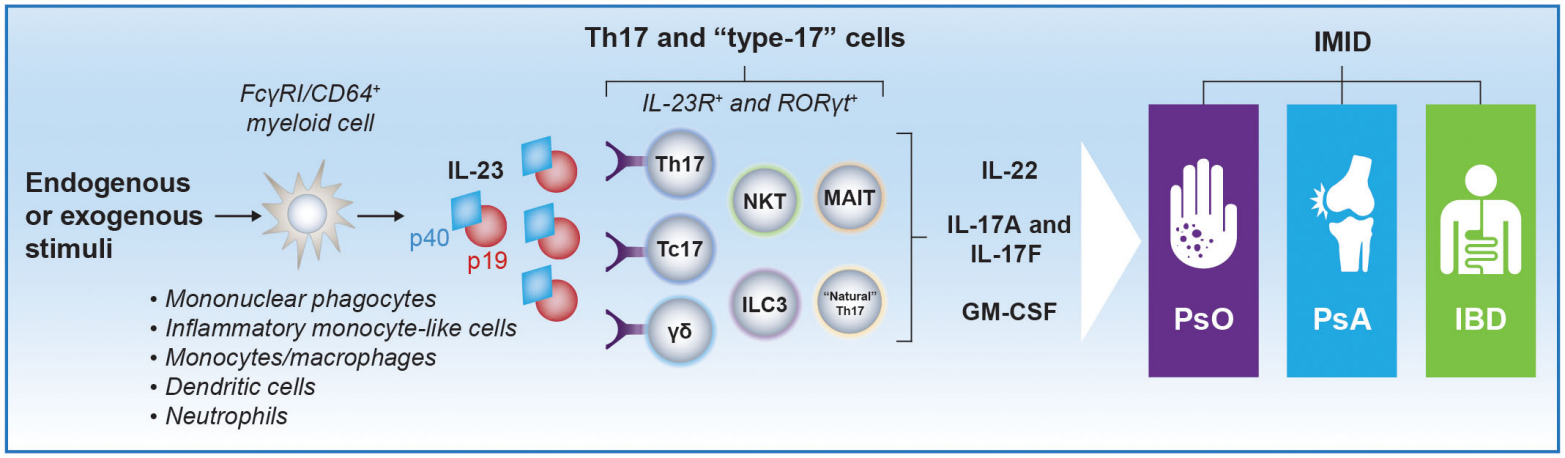
Schematic representation of IL-23–producing cells and IL-23 target cells implicated in PsO, PsA, and IBD pathogenesis. Myeloid cells are considered major producers of IL-23 in response to endogenous (eg, IL-6, TGF-β) or exogenous (eg, inflammation, infection) stimuli. Studies assessing the precise phenotypic and transcriptomic signature of IL-23–producing myeloid subsets in different IMIDs are ongoing. However, monocytes/macrophages, mononuclear phagocytes, and inflammatory monocyte–like cells that express FcγRI/CD64 have been identified as IL-23–producing cells in patients with psoriatic disease and IBD (9, 10). Semimature dendritic cells and neutrophils, which may also express FcγRI/CD64, have been identified as producers of IL-23 in PsO and IBD, respectively, as well (11, 12). High local concentrations of IL-23 produced by these cells can promote proliferation and survival of Th17 cells and stimulate other immune cells that express the IL-23 receptor and RORγt, including γδ T cells, NKTs, “natural” Th17 cells, Tc17 cells, and ILC3s, which collectively are termed type-17 cells (2). Th17 and type-17 cells induce local tissue inflammation via release of IL-17A, IL-17F, IL-22, and GM-CSF. Notably, induction of IL-17A and IL-17F production from Th17 cells does not require IL-23R signaling, but activation of the IL-23 receptor is required for pathogenic effector Th17 cell responses. FcγRI, Fc-γ receptor I; GM-CSF, granulocyte macrophage colony-stimulating factor; IBD, inflammatory bowel disease; IL, interleukin; IL-23R, IL-23 receptor; ILC, innate lymphoid cell; IMID, immune-mediated inflammatory disease; MAIT, mucosal-associated invariant T cell; NKT, natural killer T cell; PsA, psoriatic arthritis; PsO, psoriasis; RORγt, retinoic acid receptor–related orphan receptor-γt; Tc17, IL-17^+^ CD8^+^ T; Th, T helper.

In response to tissue injury or pathogenic insults, IL-23 is produced by tissue-resident myeloid cells and promotes expansion and survival of a subset of T lymphocytes, known as T helper (Th)17 cells, that produce and secrete IL-17A and other inflammatory mediators (**Figure 1**) (1, 13). Besides promoting proliferation and maintenance of Th17 cells, the cell type that has been studied most extensively with regard to IL-23 function, IL-23 can stimulate “natural” Th17 cells and IL-17^+^CD8^+^ T (Tc17) cells, as well as innate immune cells, such as γδ T cells, natural killer T cells (NKTs), mucosal-associated invariant T cells (MAITs), and IL-17–secreting innate lymphoid cells (ILC3s), all of which may be collectively termed type 3 immune cells or type-17 cells (2, 14, 15). This set of innate immune cells express the IL-23 receptor and retinoic acid receptor–related orphan receptor-γt (RORγt). The IL-23 receptor is also expressed on a small subset of interferon-γ (IFN-γ)–producing Th1 cells, where it also plays a critical role in inducing a colitogenic phenotype with induction of genes that have been implicated with human IBD by genome-wide association study (GWAS) (16). IL-23 stimulation of Th17 and type-17 cells induces local tissue inflammation via release of proinflammatory cytokines, such as IL-17A, IL-17F, IL-22, human granulocyte macrophage colony-stimulating factor (GM-CSF), IFN-γ, and tumor necrosis factor (TNF)-α. IL-23 can also suppress regulatory T (T_reg_) cell differentiation and promote expansion of tissue-resident memory T (T_RM_) cells, which have been linked to the chronicity of autoimmune and inflammatory diseases (9). IL-23 promotes programming of pathogenic Th17 cells that drive immune-mediated disease through the induction of genes encoding IL-23–dependent transcription factors, including the RORγt gene and the IL-23 receptor gene (*IL23R*), thus amplifying IL-23 signaling in a positive feedback loop and stabilizing production of proinflammatory effector molecules (17–19).

Th17 effector cytokines drive inflammation in psoriatic disease (ie, PsO and PsA) and contribute to the development of spondyloarthritis (20). In IBD, IL-23 is one of the primary drivers of inflammation, although patients with CD and UC may have different cytokine expression profiles (21). Targeting IL-23 for the treatment of psoriatic disease and IBD may restore immune homeostasis and could have significant long-term anti-inflammatory effects. Inhibition of IL-23 reduces levels of IL-17A, IL-17F, and IL-22, and markedly improves clinical outcomes for patients with these IMIDs (1, 3–7, 22, 23). Moreover, robust joint and skin responses have been sustained long-term in patients with psoriatic disease receiving anti–IL-23 therapy (24–28). Although most patients respond to treatment, some show only a partial response. Furthermore, the therapeutic benefit of IL-23 inhibition beyond this immediate cluster of IMIDs is unclear. An attenuated effect with IL-23 inhibition in some cases may be due, in part, to the timing of therapeutic intervention over the course of disease and/or limitations of currently available anti–IL-23 therapies. Also, IL-23 may be a dominant regulatory cytokine in only type 3 IMIDs driven primarily by type 3 immune cells, pathogenic type-17 cells, and/or dysregulation of the IL-23/IL-17 axis. Additional studies are needed to establish a novel molecular ontology centered around IL-23–driven disease, advance new approaches to treating inflammatory diseases with IL-23 inhibition, and ultimately identify IMID patients who represent optimal candidates to receive treatment with anti–IL-23 therapy.

## 2 Discovery of the IL-23 pathway and its pleiotropic functions

**Figure 2:**
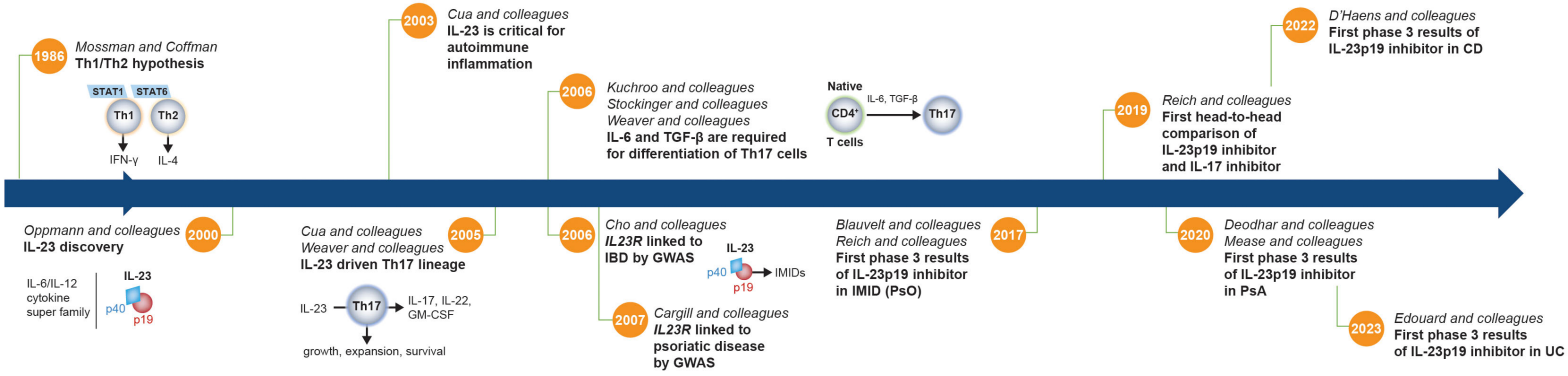
Timeline of critical discoveries related to the IL-23 pathway. Prior to the discovery of IL-23, the accepted paradigm proposed to divide CD4^+^ Th cells into 2 subsets (IFN-γ–producing Th1 cells and IL-4–producing Th2 cells) to explain the tendency of T cells to diverge into those that drive either a cellular or humoral immune response (29). However, this binary hypothesis did not explain the full spectrum of immune responses. IFN-γ–producing Th1 cells and IL-4–producing Th2 cells are dependent on STAT1 and STAT6, respectively, which did not adequately explain the regulation of STAT3-dependent CD4^+^ T cells in autoimmunity and infection. In addition, neutrophil recruitment and activation are major hallmarks of inflammation, and Th1 and Th2 cells do not promote significant neutrophil-mediated tissue injury. Following its discovery (30), IL-23 was shown to be essential for development of experimental autoimmune encephalomyelitis and was implicated in the growth, expansion, and survival of a subset of pathogenic T lymphocytes characterized by the production of IL-17A, IL-17F, IL-6, and TNF that is distinct from Th1 and Th2 cells, now known as Th17 cells (31, 32). TGF-β and IL-6 were later shown to be required to facilitate differentiation of naive T cells into Th17 cells (33–35), and the IL-23 signaling pathway was then linked to IMIDs by GWAS that identified variants of the *IL23R* gene that are associated with IBD and psoriatic disease susceptibility (36, 37). A little more than a decade later, the first phase 3 clinical trial results showing efficacy of an IL-23p19 subunit inhibitor (guselkumab) in an IMID (PsO) were published, followed over the next several years by results in PsA, CD, and UC (3–7, 38). CD, Crohn’s disease; GM-CSF, granulocyte macrophage colony-stimulating factor; GWAS, genome-wide association study; IBD, inflammatory bowel disease; IFN-γ, interferon-γ; IL, interleukin; IL23R, IL-23 receptor gene; IMID, immune-mediated inflammatory disease; PsA, psoriatic arthritis; PsO, psoriasis; STAT, signal transducer and activator of transcription; TGF-β, transforming growth factor-β; Th, T helper; UC, ulcerative colitis.

**Figure 3:**
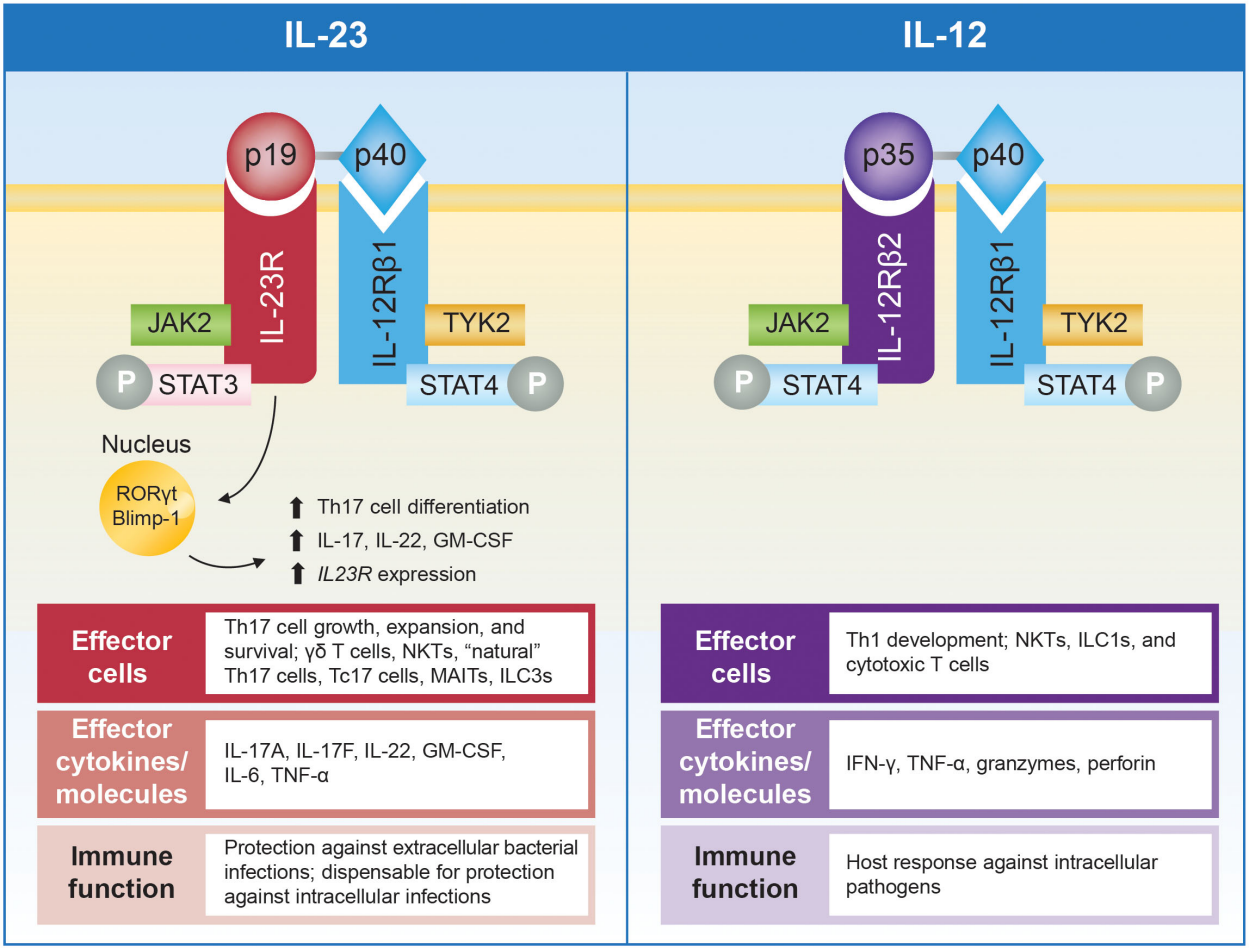
Schematic representation of IL-23 and IL-12 and their receptors, with summary of associated effector cells, cytokines, and immune function. IL-23 comprises the p19 and p40 subunits that bind to the IL-23R and IL-12Rβ1 subunits of the IL-23 receptor, respectively. IL-12 also contains the p40 subunit, which pairs with the p35 subunit that binds to IL-12Rβ2 (39). Engagement of the IL-23 receptor results in formation of a cytokine-receptor ternary complex that includes IL-23, IL-23R, and IL-12Rβ1 (40). Formation of this ternary complex activates a range of JAKs, including JAK2 and TYK2, which associate with intracellular domains of the receptor subunits and initiate specific signaling cascades (40–42). IL-23 activates pathogenic functions of CD4^+^ Th17 cells and stimulates other effector cells, including γδ T cells, NKTs, “natural” Th17 cells, Tc17 cells, MAITs, and ILC3s, that collectively produce IL-17A, IL-17F, IL-22, GM-CSF, IL-6, and TNF-α (32, 41). In contrast, IL-12 promotes differentiation of Th1 cells and activates NK cells, ILC1s, and cytotoxic T cells leading to production of IFN-γ, TNF-α, granzymes, and perforin (41). In addition, IL-23 activation of transcription factors, including STAT3, RORγt, and Blimp-1, enhances *IL23R* expression and, in a positive-feedback loop, can amplify IL-23 signaling (39, 43). RORγt represents a particularly notable IL-23–induced transcription factor essential for differentiation of naive T cells into Th17 cells. Simultaneous priming with TGF-β, IL-6, and IL-1 activates RORγt and induces expression of the IL-23 receptor, thus amplifying IL-23 signaling, which is critical for maturation and stabilization of the proinflammatory Th17 phenotype (18, 34, 35). The IL-23/Th17 pathway is important for protection against extracellular bacterial and fungal infections, while it appears to be dispensable for protection against most intracellular infections. In contrast, the IL-12/Th1 pathway is required for host responses to intracellular pathogens (44). Blimp-1, B lymphocyte–induced maturation protein 1; GM-CSF, granulocyte macrophage colony-stimulating factor; IFN, interferon; IL, interleukin; IL-12Rβ1, IL-12 receptor, β1 subunit; IL-12Rβ2, IL-12 receptor, β2 subunit; IL-23R, IL-23 receptor subunit; ILC, innate lymphoid cell; JAK, Janus kinase; MAIT, mucosal-activated invariant T cell; NKT, natural killer T cell; P, phosphoryl group; RORγt, retinoic acid receptor–related orphan receptor-γt; STAT, signal transducer and activator of transcription; Tc17, IL-17^+^ CD8^+^ T; Th, T helper; TNF-α, tumor necrosis factor-α; TYK, tyrosine kinase.

In 2000, Oppmann and colleagues identified IL-23, a novel member of the IL-12 family of cytokines comprising p19 and p40 subunits (**Figure 2**) (30). Although the p40 subunit is common to both, IL-23 (p19/p40) and IL-12 (p35/p40) are functionally distinct, with divergent roles in driving differentiation and maintenance of Th17 and Th1 cells, respectively (**Figure 3**) (1, 39, 41). While early studies using *p40^-/-^* mice and anti-p40 antibodies could not discriminate between IL-12 and IL-23, later studies in *p19^-/-^* (IL-23 deficient), *p35^-/-^* (IL-12 deficient), and *p40^-/-^* (IL-12 and IL-23 deficient) mice revealed that IL-23 was a critical cytokine driving inflammation in several models of IMIDs and exhibited functions that were pathogenetically discrete from IL-12 (1, 31, 39, 41, 45). In contrast to the role of IL-23 in driving inflammation in the skin, IL-12 receptor–mediated signaling appears protective in PsO (46). In some models of gastrointestinal inflammation, IL-12 has been shown to have a limited role, whereas other models suggest IL-12 is necessary to trigger inflammation, with IL-23 driving disease chronicity (1, 47). The roles of p19 heterodimeric cytokines paired with other subunits are not fully understood in IMIDs (1, 41).

The IL-23/IL-17 axis is also involved in host defense against certain bacterial and fungal pathogens (2) and also plays a key role in gut tissue homeostasis by supporting barrier function, tissue repair, and maintenance (1, 48). IL-17A signaling contributes to maintenance of gut epithelial homeostasis and promotes neutrophil effector function to protect against certain mucosal infections. By corollary, IL-17A inhibition has been shown to have paradoxical effects in the gut and may induce or worsen bowel inflammation (48).

The role of IL-23 in maintaining barrier homeostasis in the skin and joints is less well understood. Keratinocytes from normal skin express low levels of IL-23 (49), but it is only in the context of inflammatory skin disease that IL-23 can stimulate IL-20 family members, resulting in TNF-regulated epidermal thickening (17, 50). In the gut, epithelial-like C-X3-C motif chemokine receptor 1(CX3CR1)^+^ macrophages are an important source of IL-23 and protect from effacing bacteria, likely via modification of gastrointestinal barrier function (51). In joints, CX3CR1^+^ macrophages play a role in maintaining tight junction-mediated barriers at the synovium lining, but how IL-23 may contribute to maintaining barrier function has not yet been elucidated (52). Only a small number of IL-23–responsive cells appear to be present in entheseal tissue from mice and humans (20, 53). In response to endogenous (eg, IL-6, TGF-β) or exogenous (eg, microbial-component lipopolysaccharides, bacterial infection) stimuli, IL-23 expression increases and activates IL-23 receptor–expressing cells to produce downstream effector cytokines, contributing to local tissue inflammation that plays a role in barrier homeostasis (1, 13).

## 3 IL-23 signaling pathways

The IL-23 receptor (**Figure 3**) is constitutively expressed on the surface of NKTs, ILCs, γδ T cells and MAITs, all of which recognize antigens via invariant T-cell receptors or other motifs (42). Engagement of the IL-23 receptor results in formation of a cytokine-receptor ternary complex that includes IL-23, IL-23R, and IL-12Rβ1 (40), and activates a range of Janus kinases (JAKs; eg, JAK2, TYK2) and signal transducers and activators of transcription (STATs; eg, STAT3, STAT4). In particular, TYK2 and STAT3 activation represent key proximal signaling events for upregulating production of cytokines (eg, TNF-α, IL-17, IL-6, IL-23) implicated in IMIDs (40–42). Therefore, blockade of JAKs, including TYK2, has emerged as a therapeutic target in IMIDs (2). However, JAK inhibitors broadly inhibit the signaling of multiple cytokines and are not specific to IL-23 activation.

Induction of IL-17A and IL-17F production by Th17 cells does not require IL-23R signaling, but activation of the IL-23 receptor is required for pathogenic effector Th17 cell responses, which include production of IL-17A, IL-17F, IL-22, GM-CSF, and IFN-γ (54, 55). Type-17 cells have been shown to be important in the pathogenesis of IMIDs, with Th17 cells being major producers of IL-17A in host defense (2). There is growing evidence that two different types of Th17 cells exist *in vivo* in murine models: non-pathogenic (homeostatic) and pathogenic Th17 cells (54). Nonpathogenic Th17 cells regulate barrier functions and control microbial invasion at mucosal surfaces. Pathogenic Th17 cells induce tissue inflammation and autoimmunity. IL-23 receptor expression differentiates pathogenic from nonpathogenic type 17 cells, with pathogenic cells demonstrating high levels of IL-23 receptor expression (16). IL-21 and IL-12 were shown to stimulate IL-23 receptor expression and, therefore, promote differentiation and activation of type 17 cells *in vitro*. In addition, the localization pattern of IL-23 receptor–positive cells within tissues, particularly in barrier tissues that have resident type-17 cells, may determine disease-specific patterns of clinical inflammation when the IL-23 pathway is dysregulated (42).

The roles of IL-17, IL-22, and other effector cytokines downstream of IL-23 vary by disease state (56, 57). Cytokine expression is dynamic, and expression patterns change over the course of disease; in particular, timing and location are important. The IL-17A receptor subunit (IL-17RA), which binds IL-17A, IL-17F, IL-17A/F, and IL-25 (IL-17E), is ubiquitously expressed by a variety of cell types, including endothelial cells, keratinocytes, and gut enterocytes. Activation of these cells by IL-17A and IL-17F, usually working in synergy with other activator cytokines, promotes the expression of IL-1, IL-6, IL-8, and TNF, which may induce and sustain chronic inflammatory processes. IL-23 also regulates production of IL-22 by Th17 cells, Th22 cells, and ILC3s; furthermore, CD8^+^ T cells, γδ T cells, and NKTs can produce IL-22 in the presence of IL-23 (56). In addition, a small subset of Th1 cells was shown to express IL-23R and activation of IL-23 resulted in the induction of multiple genes that are not typically expressed in Th17 cells, but which have been linked to IBD by GWAS analysis (16). IL-17 upregulates expression of genes encoding immune chemoattractants whereas IL-22 downregulates expression of genes needed for keratinocyte differentiation; both IL-17 and IL-22 can induce expression of antimicrobial protein-encoding genes (58). IL-22 has been implicated in promoting inflammation and the pathogenesis of several diseases (eg, PsO and select cancers) but plays a protective role in other conditions (eg, asthma and pancreatitis) (56). The potentially conflicting roles for IL-22, coupled with a lack of efficacy for IL-22 inhibition in studies in some disease states, raise questions about the therapeutic utility of targeting this cytokine.

## 4 The role of the IL-23 pathway in immune-mediated inflammatory diseases

### 4.1 PsO and PsA

In **Tables 1**, **2**, we summarize the extensive experimental and clinical data characterizing the role of IL-23 and the therapeutic effect of targeting the IL-23p19 subunit in PsO and PsA. IL-23 plays an important role in the pathogenesis of psoriatic disease, in part, by contributing to recurrence of skin lesions and inducing enthesitis in PsO and PsA, respectively (20, 63, 90). Clinical data suggest that selective IL-23p19 subunit inhibitors are effective in psoriatic disease. In PsO, targeting the IL-23p19 subunit can restore favorable ratios of T-cell populations in lesional skin compared with IL-17A inhibition (9). In PsA, targeting the IL-23p19 subunit can modulate expression of disease-associated genes, with directionality toward normalization of whole blood transcriptomes towards those found in healthy volunteers (87). Selective blockade targeting of the IL-23p19 subunit has not elicited specific safety signals in either PsO or PsA (5, 6, 68, 71, 84, 85). The clinical efficacy of selective IL-23 inhibition has been established, with anti–IL-23 therapy approved for use in both PsO and PsA. In addition, head‐to‐head comparative data from clinical trials of patients with PsO suggest that IL-23p19 subunit inhibitors (ie, guselkumab, risankizumab) have superior efficacy over the TNF inhibitor adalimumab (72), the IL-17A inhibitor secukinumab (68, 71), and the IL-12/IL-23 inhibitor ustekinumab (91). Nevertheless, onset of efficacy with IL-23p19 subunit inhibitors has been shown to be less rapid than with IL-17A inhibitors, as observed in the head-to-head comparison of guselkumab and ixekizumab for PsO (71, 75). In PsA, there are no head-to-head comparisons of IL-23p19 subunit inhibitors versus other targeted PsA therapies. A systematic literature review and network meta-analysis of clinical trials in patients with PsA showed that IL-23p19 subunit inhibition with guselkumab provided better skin efficacy than most TNF and JAK inhibitors, while demonstrating joint efficacy comparable to IL-17A, JAK, and TNF inhibitors administered by subcutaneous injection (92).

**Table 1:** PsO and IL-23.

	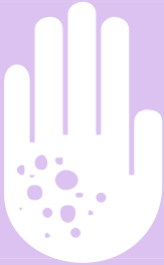
	PsO
Disease description	• Chronic, relapsing-remitting disease characterized by well-demarcated red, scaly plaques (59) • Multifactorial etiology, including immune system dysregulation, genetic susceptibility, and environmental triggers (59, 60) • Both keratinocytes and immune cells produce and respond to cytokine mediators of inflammation, resulting in amplification of immune responses via a feedforward mechanism (61) • Considered a systemic inflammatory disorder associated with serious comorbidities, including PsA, IBD, and cardiovascular disease (59)
Genetic studies	• Protective SNPs in the *IL12B* and *IL23R* genes have been found in significantly lower proportions of patients when compared with matched controls (37) • As of 2020, over 80 genetic loci associated with PsO susceptibility have been identified; specific genes include *IL23R*, *LCE3A*, *LCE3D*, *TRAF3IP2*, HLA-A*02:07 (62) • HLA-C*06:02, located at the *PSORS1* locus, may account for up to 50% of disease heritability and is associated with enhanced clinical responses to IL-12/IL-23p40 inhibition (59)
Role of IL-23	• FcγRI/CD64^+^ monocytes/macrophages and mononuclear phagocytes have been identified as major IL-23–producing cells in lesional skin of patients with PsO; semimature dendritic cells, which may also express FcγRI/CD64, have been identified as major producers of IL-23 as well (9) • PsO is characterized by activation of innate and adaptive IL-23–driven Th17 cell and type-17 cell responses and cytokine production (IL-17A, IL-17F, and IL-22) (32) • Tc17 cells localized in the epidermis may be key pathogenic cells (11) • Activated type-17 cells make IL-17A or IL-17F individually or together, which can create differing effects on affected tissues. Type-17 cells vary in their expression of IFN-γ and IL-10, expressing either or both (11) • IL-23 can also promote expansion of T_RM_ cells, which are linked to recurrence of lesions and chronicity of disease (63) • Activation of cytokines and signaling downstream of IL-23 induces keratinocyte proliferation, increases expression of mediators of angiogenesis and endothelial adhesion molecules, and promotes infiltration of immune cells into lesional skin (60)
IL-23 inhibition	• Guselkumab, risankizumab, and tildrakizumab are selective IL-23p19 subunit inhibitors currently approved for treatment of moderate to severe plaque PsO • High levels of clinical response may be achieved with IL-23 inhibition and sustained through up to 5 years of treatment (3, 4, 25, 26, 64–67) • Durability of clinical response is observed following withdrawal of IL-23 inhibitors, an effect that is associated with continued suppression of the IL-23 axis (68–70) • Selective IL-23 inhibition has also been shown to favorably shift the relative ratio of CD8^+^ T_RM_ cells and T_reg_ cells in lesional skin compared with IL-17A inhibition (9). Inhibition of IL-23 is hypothesized to lead to long-lasting therapeutic effects by suppressing T_RM_ cells, which might not be observed through blockade of an effector cytokine, such as IL-17A • Specific safety signals associated with direct IL-17A inhibition (eg, *Candida* infections, IBD) were not observed with IL-23p19 subunit inhibitors in PsO studies (68, 71)
Comparison to other targeted therapies	• Selectively targeting the IL-23 pathway provides a higher degree of efficacy versus TNF-α blockade, with more complete inhibition of PsO-specific cytokines (3, 4, 64, 72) • Better efficacy has been observed with most IL-23p19 subunit inhibitors versus IL-12/IL-23p40 inhibition (65, 73). IFN-γ inhibition has been shown to have minimal efficacy in the treatment of PsO, suggesting a more limited role of the IL-12/Th1 axis in PsO pathogenesis (74) • Current evidence suggests that targeting the IL-23p19 subunit is superior to IL-17A inhibition in achieving long-term clinical responses (48 or 52 weeks) (68, 71) • Head‐to‐head comparison data suggest that patients treated with ixekizumab (an IL-17A inhibitor) achieve skin clearance more rapidly than patients treated with guselkumab (75)

FcγRI, Fc γ receptor I; HLA, human leukocyte antigen; IBD, inflammatory bowel disease; IFN-γ, interferon-γ; IL, interleukin; LCE3A, late cornified envelope 3A; LCE3D, late cornified envelope 3D; PsA, psoriatic arthritis; PsO, psoriasis; SNP, single nucleotide polymorphism; Tc17, IL-17^+^ CD8^+^ T; Th, T helper; TNF-α, tumor necrosis factor-α; TRAF3IP2, TNF receptor–associated factor 3 interacting protein 2; T_reg_, regulatory T; T_RM_, tissue-resident memory T.

**Table 2:** PsA and IL-23.

	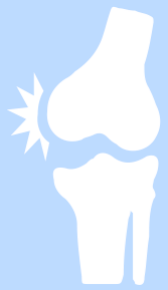
	PsA
Disease description	• Spondyloarthropathies are a family of interrelated inflammatory rheumatic diseases with common characteristics, including axial inflammation, peripheral oligoarthritis, enthesitis, a link to infection or intestinal dysfunction, and seronegative status for RF (76) • Patients with PsA are seronegative for RF and CCP antibodies; only 40% of patients have increased CRP and/or ESR (77) • Radiologically, PsA may be characterized by bone and cartilage destruction with pathologic new bone formation • Gut inflammation is an important risk factor driving spondyloarthropathies, including PsA (78) • On average, onset of PsA occurs 10 years after initial presentation with PsO, and PsA is associated with both enthesitis and dactylitis
Genetic studies	• Susceptibility to spondyloarthropathies and related conditions is associated with IL-23 pathway–related gene polymorphisms, including those in the genes encoding IL-23A and IL-23 receptor, JAK2 and TYK2, STAT3, and IL-17RA (76, 79) • HLA-B27, a ubiquitous MHC Class I molecule, is associated with spondyloarthropathies, and approximately 25% of patients with PsA are positive for HLA-B27 (77) • Studies conducted in HLA-B27 transgenic rats indicate possible links between HLA-B27 and misfolding, ER stress, and augmented induction of the IL-23 pathway (80)
Role of IL-23	• IL-23/IL-23 receptor– and IL-17/IL-17 receptor–expressing cells can be found in synovial fluid, and IL-23–induced signaling can affect pannus formation, joint erosion, and osteoclastogenesis (81, 82) • IL-23 has been shown to drive enthesitis and stimulate entheseal CD3^+^ lymphocyte-dependent secretion of IL-17 and IL-22, inducing enthesitis and new bone formation (20) • Human entheseal tissue has been shown to contain IL-23–expressing ILC3s that may have a role in the pathogenesis of spondyloarthropathies (83)
IL-23 inhibition	• Guselkumab and risankizumab are IL-23p19 subunit inhibitors approved to treat active PsA; tildrakizumab, another IL-23p19 subunit inhibitor, is being studied in PsA clinical trials • IL-23p19 subunit inhibitors were superior versus placebo in clinical trials for PsA; clinical response was sustained with up to 2 years of treatment (5, 6, 25, 26, 66, 67, 84–86) • Significantly better resolution of enthesitis and dactylitis was observed with guselkumab or risankizumab versus placebo, but not tildrakizumab • IL-23p19 subunit blockade normalizes the whole blood PsA disease–associated gene expression profile (87) and disrupts serum collagen biomarker profiles (88) • Specific safety signals associated with direct TNF, JAK, and IL-17 inhibition were not observed in clinical trials with selective IL-23p19 subunit inhibitors in PsO and PsA (5, 6, 84, 85, 89) • Additional studies are warranted to help answer remaining questions regarding the efficacy of IL-23p19 subunit inhibitors in PsA (eg, whether clinical response rate is limited by tissue penetration of IL-23p19 subunit inhibitors)
Comparison to other targeted therapies	• Head-to-head trials comparing IL-23p19 subunit inhibitors versus other biologics for PsA have not been conducted

CCP, cyclic citrullinated peptide; CRP, C reactive protein; ER, endoplasmic reticulum; ESR, erythrocyte sedimentation rate; HLA, human leukocyte antigen; IBD, inflammatory bowel disease; IL, interleukin; ILC, innate lymphoid cell; JAK, Janus kinase; MHC, major histocompatibility complex; PsA, psoriatic arthritis; PsO, psoriasis; RF, rheumatoid factor; SNP, single nucleotide polymorphism; STAT, signal transducer and activator of transcription; TNF-α, tumor necrosis factor-α; TYK, tyrosine kinase.

### 4.2 Axial spondyloarthritis

Despite experimental evidence from animal models that suggests a pathogenic role for IL-23 in axial spondyloarthritis, clinical trials of IL-23p19 subunit and IL-12/IL-23p40 subunit inhibitors in patients with axial spondyloarthritis failed (93, 94). The lack of efficacy of IL-23 blockade in axial (axial spondyloarthritis) but not peripheral (PsA) spondyloarthritis may be due to differences in IL-23 responsive cell types involved in disease pathogenesis and the timing of therapeutic intervention in the course of these diseases (95). Limited penetration of large molecule therapeutic antibodies into joint tissues, such as synovium, may also limit efficacy. In contrast to trials in axial spondyloarthritis, *post hoc* analyses of selective IL-23p19 subunit inhibition with guselkumab in patients with axial PsA showed significant improvement in symptoms of axial involvement versus placebo (96). A prospective, controlled trial of guselkumab in patients with PsA with magnetic resonance imaging–confirmed axial involvement is ongoing to further expand upon this finding (ClinicalTrials.gov Identifier: NCT04929210).

### 4.3 IBD

In **Table 3**, the genetic, experimental, and clinical evidence supporting a role for IL-23 inhibition in IBD is summarized. In inflamed tissue, overexpression of IL-23 converts nonpathogenic Th17 cells into pathogenic effectors, resulting in impaired barrier function, and drives continued inflammation via downstream pathways, leading to survival and expansion of Th17 cells (1). Moreover, IL-23 signaling can suppress T_reg_ cell differentiation, a cell population that is important in regulation and suppression of chronic gut inflammation (1). Risankizumab is an IL-23p19 subunit inhibitor approved for use in many countries for CD, while mirikizumab is an IL-23p19 inhibitor approved for treatment of UC in Japan and by the European Medicines Agency. IL-23p19 subunit inhibitors have generally demonstrated efficacy in clinical and/or endoscopic outcomes, with evidence to support maintenance of long-term clinical responses and a favorable safety profile (7, 8, 101, 102, 104, 105). One systematic review and network meta-analysis of clinical trials for biologic therapies for CD suggested that IL-23p19 subunit inhibition with risankizumab may yield better outcomes compared to other biologics and be preferred as second-line therapy for induction of clinical remission (106). However, head-to-head comparative studies of IL-23p19 subunit inhibitors versus other biologics for IBD are warranted to evaluate the relative efficacy of IL-23p19 subunit inhibitors versus other biologics.

**Table 3:** IBD and IL-23.

	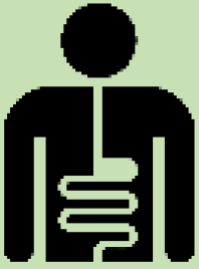
	IBD
Disease description	• Typically categorized as CD or UC; characterized by chronic inflammation of the intestinal tract (97) • Genetic and environmental factors, as well as dysregulated interplay between the gut microbiota, epithelial-mucosal barrier, and the intestinal immune system in susceptible individuals, are believed to play a role in the pathogenesis of IBD (97, 98) • When the integrity of the epithelial-mucosal barrier is compromised, normally innocuous commensal bacteria may become pathogenic by crossing the epithelium and eliciting an immune response and intestinal inflammation (97) • Inflammation in CD is characterized by patchy, transmural inflammation anywhere throughout the gastrointestinal tract, while UC typically shows continuous, more superficial inflammation of the mucosa and submucosa that is usually restricted to the colon (97) • The most common presenting symptoms of UC and CD include increased frequency of defecation and diminished stool consistency (diarrhea) (99) • Considered systemic diseases, with patients frequently suffering from extraintestinal manifestations and inflammatory comorbidities involving the skin, eyes, or joints (99)
Genetic studies	• Strong associations have been observed between IL-23 receptor polymorphisms and susceptibility to CD (36) • IL-23 receptor polymorphisms have also been associated with UC (100)
Role of IL-23	• IL-23 is expressed in healthy gut, but IL-23 expression is higher in patients with IBD compared with healthy controls (41) • FcγRI/CD64^+^ monocyte/macrophage and inflammatory monocyte-like cells have been identified as major IL-23–producing cells in patients with IBD; neutrophils, which may also express FcγRI/CD64, have been identified as major producers of IL-23 as well (10, 12) • Overexpression of IL-23 is associated with impaired barrier function and drives continued inflammation via downstream pathways, leading to differentiation, survival, and expansion of Th17 cells (1) • Although the interplay of immune factors is complex, certain cytokines, including IL-23, and innate/adaptive immune pathways are particularly important regulators of gut inflammation (1, 47) • T_reg_ cells are important in the regulation and suppression of chronic inflammation, and their numbers are reduced in IBD. IL-23 signaling has been shown to suppress T_reg_ cell differentiation (1)
IL-23 inhibition	• Risankizumab is approved for the treatment of CD; mirikizumab is an IL-23p19 inhibitor approved for treatment of UC in Japan and by the European Medicines Agency • IL-23p19 subunit inhibitors being studied for the treatment of CD and/or UC include guselkumab, risankizumab, and mirikizumab • IL-23p19 subunit inhibitors demonstrated efficacy in clinical and/or endoscopic outcomes with induction treatment in patients with CD and UC (7, 8, 101, 102) • Although long-term data are currently limited, clinical responses were maintained in patients with CD and UC for up to 52 weeks with continued IL-23p19 subunit inhibitor treatment (8, 101) • Endoscopic remission and response observed with IL-23p19 subunit inhibitors in patients with IBD (CD) are associated with significant transcriptomic changes in the colon. In addition, 12 weeks of IL-23p19 subunit inhibitor treatment had differential effects on expression of genes associated with the IL-23/IL-17 axis in the colon and ileum (22) • The safety profile of IL-23p19 subunit inhibitors in IBD is similar to that observed in other IMIDs (7, 8, 101–103)
Comparison to other targeted therapies	• Head-to-head clinical trials of IL-23p19 subunit inhibitors versus other biologics for IBD have not been conducted; thus, more clinical evidence is warranted to support the efficacy of IL-23p19 subunit inhibitors compared to other agents in the same class or in other classes

CD, Crohn’s disease; FcγRI, Fc-γ receptor I; IBD, inflammatory bowel disease; IL, interleukin; IMID, immune-mediated inflammatory disease; Th, T helper; T_reg_, regulatory; UC, ulcerative colitis.

### 4.4 Other inflammatory diseases

Other IMIDs in which IL-23 has been implicated through genetic association, tissue expression, animal models, and plausible biological profile include palmoplantar pustulosis, pityriasis rubra pilaris, rheumatoid arthritis, multiple sclerosis, giant cell arteritis, and type 1 diabetes (31, 45, 107–118). Guselkumab is an IL-23p19 subunit inhibitor approved for treatment of palmoplantar pustulosis in Japan and other Asian countries. Selective targeting of the IL-23p19 subunit improved clinical outcomes for patients with palmoplantar pustulosis and a small number of patients with pityriasis rubra pilaris (107, 118).

In contrast, IL-12/IL-23p40 subunit inhibition failed to show clinical benefit in multiple sclerosis, and IL-12/IL-23p40 or IL-23p19 subunit-targeted therapy failed to improve clinical outcomes in studies of patients with active rheumatoid arthritis, despite strong scientific rationale for this approach derived from animal models (31, 45, 108, 110). The IL-23/IL-17 axis has been implicated in driving intrinsic inflammatory activity and triggering the clinical onset of rheumatoid arthritis, primarily in animal models, suggesting that early intervention in the pre–rheumatoid arthritis phase could potentially lead to clinical improvement (111, 119). Observations in animal models of multiple sclerosis suggest that IL−23 is necessary for the induction of disease but not disease maintenance, and that lack of IL−23 is not protective once encephalitogenic T cells have developed (112). Patients with multiple sclerosis may therefore potentially benefit from targeting the IL-23/IL-17 axis at an earlier stage of disease. Treatment of multiple sclerosis is further complicated by the need for therapeutic agents that cross the blood-brain barrier (120). Nevertheless, IL12/23p40–targeted therapy in patients with established multiple sclerosis was ineffective (121), whereas the impact of IL-23p19 subunit inhibition in this patient population has yet to be determined. However, the anti–IL-17A therapeutic antibody secukinumab was shown to decrease the development of new lesions in a small, double-blind clinical trial of patients with multiple sclerosis (122).

Conflicting results regarding the efficacy of IL-12/IL-23p40–targeted therapy in patients with giant cell arteritis have been observed across trials, and further studies are needed for more definitive evidence (117, 123). A proof-of-concept study to evaluate targeting the IL-23p19 subunit with guselkumab in patients with new-onset or relapsing giant cell arteritis is ongoing (NCT04633447). IL-12/IL-23p40 inhibition is currently being assessed for treatment of new-onset type 1 diabetes in clinical studies (113, 114). IL-23 gene expression was shown to be increased in the pancreatic islet cells of a patient with type 1 diabetes, and targeting both the IL-23p19 and IL-12/IL-23p40 subunits has been shown to suppress the incidence of diabetes in nonobese diabetic mice (115, 116).

The pleiotropic nature of the biology of IL-23 also predicts its potentially important role in other diseases beyond IMIDs, such as obesity (124), atherosclerosis (125), stroke (126), cancer (1, 39), mood disorders (124), and neurodegenerative diseases (127). Although IL-23 could be involved in the pathogenesis of multiple disease states, it may act on different cell types or at different stages of disease in each, yielding nuanced effects, a wide range of clinical manifestations of disease, and varied implications of IL-23p19 subunit-targeted therapy.

## 5 Exploring the frontiers of IL-23 molecular biology

Further investigation is needed to better understand the molecular biology of IL-23 to properly adapt anti–IL-23 therapy across the breadth of IMIDs. Herein, we propose a multiomics approach to advance the science of IL-23, including comprehensive studies to identify the spectrum of IL-23 receptor–expressing cells, and to provide insights into the role of IL-23 in driving IMIDs. Moreover, we identify gaps in the understanding of IL-23 signaling and cellular activity and discuss durability of treatment response and future prospects for anti–IL-23 therapeutic intervention, including potential approaches for combination therapy (**Box 1**).

### 5.1 A multiomics approach to advance IL-23 science

The emergence of high-throughput technologies over the past 30 years has revolutionized medical research. The “omic” technologies provide a comprehensive assessment of genomics, epigenomics, transcriptomics (including single cell RNA-seq and spatial transcriptomics), proteomics, metabolomics, and microbiomics. Each of these “omic” technologies provides a global pattern of molecular signatures (genes, epigenetic factors, RNA transcripts, proteins, or metabolites) that are altered in disease, providing insight into biomarkers, pathways, or cell types involved in disease pathogenesis. Combining these “omic” technologies can provide a more complete picture of the molecular factors that drive functional consequences and interactions of IL-23 signaling underlying the pathogenesis and phenotypic manifestations of IMIDs (128–130). For instance, a recent genome-wide study identified allelic variants of the HLA-C upstream region (which includes *PSORS1C3, MICA*, and *TNFA*) that were associated with clinical response to anti-IL-12/IL-23 therapy (131). In addition, an interaction between gut microbiota composition and responsiveness to IL-17 and IL-23 inhibitors has been observed in patients with PsO (132). Another example derives from a multiomics study that used matched colonic biopsy tissue and serum samples at multiple time points from IBD patients treated with guselkumab or placebo. This application of a multiomics approach allowed deeper understanding of local (gastrointestinal tissues) versus systemic (serum protein) immune responses driven by IL-23 in IBD patients and revealed that guselkumab mainly suppressed local gastrointestinal tissue inflammation by normalization of gut epithelial cell-injury responses (manuscript in preparation).

### 5.2 An atlas of IL-23 receptor–expressing cells

A definitive atlas of IL-23 receptor–expressing cells across different tissues and at different phases of disease activity or progression in patients would be a valuable resource to further elucidate understanding of normal physiological and pathophysiological function of IL-23. For example, the role of IL-23 receptor signaling in T cells has been most extensively studied in experimental animal models, and the effect of IL-23 on diverse T-cell targets in human patients is not well understood (133). Generally, the IL-23 receptor is expressed at very low levels and is difficult to detect at the protein level; therefore, a combination of spatial transcriptomic and RNA sequencing data may elucidate IL-23 receptor–expressing cell populations of interest with high resolution. Data from single cells may then be interpreted in the context of broader sequencing data to better understand the IL-23 pathway gene expression profile. In turn, a recently developed and validated panel of anti–human IL-23 receptor antibodies is being utilized to identify and characterize unique IL-23 responding populations across inflamed skin, joint, and gut tissues (Lin et al, manuscript in preparation). These studies will complement IL-23 receptor transcriptomic analyses and contribute to further elucidating the role of IL-23 during disease pathogenesis.

### 5.3 Gaps in understanding IL-23 signaling and cellular activity

Studies on IL-23 signaling and cellular activity are needed to further characterize the normal physiological function of IL-23 and to identify new targets and strategies for drug development independent of direct IL-23 engagement in order to more fully tap into the therapeutic potential of inhibiting the IL-23 pathway. Gaps currently exist with respect to the impact of therapeutic intervention at the level of downstream effectors, potential modulators of IL-23 signaling, and the molecular basis for inadequate response to IL-23 inhibition in some cases.

Inhibiting IL-23 directly may have broader utility than targeting downstream effectors of the IL-23 pathway. For example, targeting members of the IL-17 family of cytokines may not sufficiently or uniformly suppress inflammatory pathways owing to the functional redundancy of IL-17 family cytokines in promoting inflammation (134). However, not all inflammatory diseases in which IL-17A or IL-17F are considered pathogenic drivers are necessarily IL-23–dependent (eg, hidradenitis suppurativa). As aforementioned, the effect of IL-23 on diverse T-cell targets in patients is not yet well understood. Comparing and contrasting effector and regulatory pathway mediators across diseases in patients will better describe the impact of IL-23 and, in turn, IL-23 inhibition on broader T-cell populations. Although IL-23 signaling is generally highly conserved across species, experiments using human cells or tissues are essential to ensure relevance to human disease. Approaches to characterize IL-23–producing and IL-23–responsive cells from clinical tissue samples, such as single-cell transcriptomics, will better refine understanding of IL-23 signaling pathways and provide temporal and spatial maps of IL-23 pathway dysregulation in tissues (11).

Even within the cluster of IMIDs that is sensitive to selective IL-23p19 subunit targeting, not all patients respond adequately. Additional research on signaling pathways downstream of IL-23 may determine why some patients experience inadequate response and identify potential complementary therapeutic targets. In turn, it will be important to understand synergistic and antagonistic mediators of IL-23 receptor signaling, and how they are dependent on the local tissue microenvironment. For example, in a recent study, quantitative phospho-proteomic analysis of Th17 cells was used to identify potential targets downstream of IL-23 signaling (128). Similar approaches may be used to address questions such as whether there are nuances to IL-23 signaling in different cell types or tissues that may impact responses to therapeutics targeting the IL-23p19 subunit. Furthermore, signaling crosstalk between IL-23 and other cytokines, such as IL-1β, can augment and sustain IL-23 signaling (135); study of such interactions is now feasible given the availability of high-fidelity resolution techniques. In this context, other pathogenetic components, such as the skin and gut microbiomes, may influence IL-23 receptor signaling, given its critical roles in protecting against invading pathogens, educating the immune system, and metabolizing natural products in these tissues (136).

## 6 Frontiers for exploration in therapeutic intervention through IL-23 inhibition

### 6.1 Durability of response

T_RM_ cells are a subset of noncirculating memory T cells that persist over the long-term in peripheral tissues. T_RM_ cells provide rapid, local immune responses on re-exposure to pathogens under healthy conditions, but also develop after sensitization to self-antigens and are involved in the pathogenesis of autoimmune disorders. T_RM_ cells are a key reason for why some IMIDs, such as PsO, are chronic, life-long diseases, and why skin lesions in PsO tend to recur at the same sites upon relapse of disease. T_reg_ cells help to maintain immune homeostasis and self-tolerance by suppressing inflammation and effector T cells. IL-23 has been shown to suppress the differentiation of T_reg_ cells (9), and promote differentiation, survival, and expansion of pathogenic Th17 cells (13).

The durable, long-lasting therapeutic effect observed with selective targeting of IL-23p19 subunit is hypothesized to derive from suppression of T_RM_ cells. IL-23 inhibition has been shown to favorably shift the relative ratio of CD8^+^ T_RM_ cells and T_reg_ cells in PsO lesions compared with IL-17A inhibition (9). Moreover, clinical studies have shown superior long-term maintenance of response with IL-23 inhibition over IL-17A blockade; IL-17A inhibitors, in essence targeting an effector cytokine, have less impact on the relative numbers of proinflammatory and anti-inflammatory T cells compared to IL-23 inhibitors (71). Nonetheless, further studies with larger numbers of samples over long-term duration of follow-up are needed to confirm these findings. Future studies are needed to determine whether selective IL-23 inhibition, if initiated in the optimal context, can result in the reduction or even elimination of pathogenic T_RM_ cells and ultimately drive PsO and other IMIDs into long-term remission (9). The intriguing possibility of prolonged drug-free remission is particularly enticing in this context. The ongoing GUIDE study is evaluating the implications of early intervention with guselkumab in patients with PsO and short disease duration to achieve a super-response, extend dosing interval, and maintain disease control after treatment withdrawal, and is further exploring the impact of guselkumab treatment on the balance of T_RM_/T_reg_ cell populations in patients with PsO over time (137).

### 6.2 Future IL-23 inhibitor molecules

Specific attributes of currently available and future IL-23 inhibitors may enhance blockade of IL-23 signaling and further improve clinical outcomes. The *in vitro* binding affinity for IL-23 and potency for blocking IL-23 signaling of available antibodies targeting the IL-23p19 subunit may be different, potentially driving differences in clinical performance of these drugs (138). However, the relevance of bioavailability and serum levels of IL-23 inhibitors to clinical efficacy is unclear given that IL-23 is produced and drives inflammation within the local tissue microenvironment. It is important to note, however, that IL-23–producing cells are also capable of trafficking to other tissue sites to extend or propagate disease pathogenesis (139). The mechanism by which an anti–IL-23 antibody localizes to inflamed tissue(s) to sequester and neutralize IL-23 at the site of its production and activity may represent an important attribute for consideration. For example, myeloid cells that express FcγRI/CD64 have been identified as major IL-23–producing cells in preclinical models as well as in lesional tissue from human patients with psoriatic disease and IBD (9, 10). Thus, anti–IL-23 monoclonal antibodies that can also bind to FcγRI/CD64 via their Fc region may be optimally localized within the inflamed tissue microenvironment to neutralize IL-23 at its cellular source and potentially provide a therapeutic advantage.

A potential alternative to directly targeting the IL-23p19 subunit is to target the IL-23 receptor itself. Orally bioavailable therapeutic peptides that bind the IL-23 receptor with high specificity and affinity offer promise (140–142). Small molecules and peptides may have an advantage over therapeutic antibodies by virtue of better tissue penetration, which could potentially improve outcomes for and expand the scope of IMIDs that are treatable through inhibition of IL-23 receptor signaling. An oral IL-23 receptor antagonist peptide (142) is currently being studied in PsO and UC in clinical trials, and future studies in other IMIDs may be warranted.

### 6.3 Combination therapy anchored on IL-23 inhibition

Although monotherapy with selective anti–IL-23 antibodies has been shown to be therapeutically effective across a variety of IMIDs, some patients fail to achieve adequate or durable responses. One approach to addressing remaining unmet needs across IMIDs is to also target complementary pathogenic pathways through combination therapy. IL-23 inhibitors may be useful in the context of combination therapy because of their potent ability to target a dominant, regulatory cytokine that serves as a rheostat to control the stream of inflammatory signals that can drive IMID pathogenesis. Moreover, the safety profile of IL-23 inhibitors has been thus far reassuring, which further supports pairing IL-23 inhibition with a second mechanism in a combination therapy approach. In turn, IL-23 blockade may synergize with inhibition of a pathway with a complementary mechanism of action, such as the TNF pathway, to achieve even higher levels of efficacy than those achievable using either approach alone, and potentially with minimal risk of compounding safety concerns, a problem that has beset prior combination therapeutic approaches for cytokine blockade.

Recently, the combination of IL-23 and TNF inhibition was shown to have a synergistic effect on preventing loss of body weight and reducing local colonic tissue inflammation in an anti-CD40 antibody–induced colitis mouse model (143). In that preclinical study, improvements in disease features were accompanied by normalization of gene expression patterns in shared molecular pathways for either treatment when used as monotherapy and when used in combination; however, normalization of expression of gene networks that were uniquely impacted in response to combination therapy was also observed. In the phase 2 VEGA study for UC, combination induction treatment with both an IL-23 inhibitor and a TNF inhibitor more effectively induced clinical response, remission, and endoscopic improvement than monotherapy with either agent alone (144). Further still, combination therapy was also shown to reverse the disease transcriptomic profile to a greater extent than either monotherapy alone (145). An additional ongoing clinical trial is studying the impact of IL-23 inhibition in combination with TNF inhibition in PsA (NCT05071664).

## 7 Discussion

Remarkable progress over the last 20 years has advanced our understanding of the science of IL-23, leading to the development of transformational therapies that selectively block IL-23. IL-23 may represent a dominant regulatory cytokine in the context of type 3 IMIDs, with broad effects mediated by regulation of Th17 cells, other IL-23 receptor^+^ type-17 cells, and/or the IL-23/IL-17 axis. Extensive experimental and clinical evidence supports a role for IL-23 as a critical regulatory cytokine in a class of IMIDs that includes PsO, PsA, and IBD. The use of IL-23 inhibitors has been very successful in treating these IMIDs. However, not all patients respond adequately to currently available anti–IL-23 therapies and some patients may show only a partial response to treatment. IL-23 inhibition has been unsuccessful in clinical trials for some IMIDs, despite strong *a priori* scientific rationale for anti–IL-23 treatment. Studies to determine the therapeutic efficacy of IL-23 inhibition in yet other inflammatory diseases are needed. Factors contributing to these observations may include the timing of therapeutic intervention over the course of disease and limitations of currently available IL-23 inhibitor therapies.

We propose additional studies to address remaining challenges and unmet needs pertaining to anti–IL-23 therapy and to advance the science of IL-23. These include studies using a multiomics approach to: 1) further define the identities and roles of IL-23–expressing cells and IL-23 receptor–expressing cell targets in different inflammatory diseases; 2) characterize nuanced effects of IL-23 in different cell types or tissues; 3) describe changes in IL-23 receptor expression and signaling over the course of disease; and 4) characterize the mechanisms underlying tissue-specific responses and durability of response with IL-23 inhibitor therapy. Results from such studies hold strong potential to establish a novel molecular ontology centered around IL-23–driven disease, enhance and/or complement therapeutic approaches for IL-23 inhibition in treating IMIDs, further understand variability in achieving clinical responses, and ultimately better identify ideal patient candidates for anti–IL-23 therapies. The future appears promising, with the emergence of new strategies, including combination therapy, and ongoing progress in the understanding of the science of IL-23 to foster the next generation of therapeutics targeting the IL-23 pathway.

## Author contributions

JK: Conceptualization, Writing – review & editing. KE: Conceptualization, Writing – review & editing. VK: Conceptualization, Writing – review & editing. CR: Conceptualization, Writing – review & editing. MA: Conceptualization, Writing – review & editing. ME: Conceptualization, Writing – review & editing. AF: Conceptualization, Writing – review & editing. SF: Conceptualization, Writing – review & editing. JS: Conceptualization, Writing – review & editing. Y-WY: Conceptualization, Writing – review & editing. DC: Conceptualization, Writing – review & editing. IM: Conceptualization, Writing – review & editing.
